# Emerging technologies in poultry genomics: Unlocking innovation for the future of sustainable production

**DOI:** 10.1016/j.psj.2025.106240

**Published:** 2025-12-10

**Authors:** Huaijun Zhou, Fiona M. McCarthy, Tae Hyun Kim, Wesley Warren, Guolong Zhang

**Affiliations:** a Department of Animal Science, University of California, Davis, Davis, CA, USA; b School of Animal & Comparative Biomedical Sciences, University of Arizona, Tucson, AZ, USA; c Department of Animal Science & The Huck Institutes of the Life Sciences, The Pennsylvania State University, University Park, PA, USA; d Division of Animal Sciences, University of Missouri, Columbia, MO, USA; e Department of Animal and Food Sciences, Oklahoma State University, Stillwater, OK, USA

**Keywords:** Functional annotation, CRISPR gene editing, Single-cell RNA sequencing, Microbiome–host interactions, Sustainable poultry production

## Abstract

Over the past decade, poultry genomics has undergone a transformative shift from genome assembly to functional annotation, mechanistic discovery, and integrative applications that advance sustainable poultry production. This symposium highlights emerging tools and resources that enable researchers to move beyond statistical associations toward biological causality and breeding application. Functional annotation through the FAANG (Functional Annotation of Animal Genomes) initiative and ChickenGTEx Project has produced comprehensive regulatory maps and expression QTL datasets across tissues, cell types, and developmental stages, forming a foundation for identifying causal regulatory variants. Advances in CRISPR-based editing provide scalable platforms for *in vitro* validation of regulatory elements and dissect gene function, bridging genotype to phenotype. Single-cell RNA sequencing technologies are now delivering high-resolution immune cell atlases and developmental maps, offering novel insights into host defense and tissue regulation. Integrative omics frameworks that combine genomics, transcriptomics, epigenomics, and 3D chromatin data are revealing systemic regulatory networks controlling complex traits such as egg production, growth, and fat deposition, with functional validation of candidate variants accelerating their translation into precision breeding. Equally important, microbiome-based approaches are emerging as powerful tools to improve poultry health, nutrient utilization, and disease resistance, providing environmentally sustainable strategies that complement genetic selection. Challenges remain in statistical power, tissue- and development-specific context specificity, and bridging discoveries to genomic prediction. However, new opportunities, including multi-omics integration, causal inference, and iterative validation pipelines facilitate the development of predictive and mechanistically informed approaches to genetic improvement. Together, these advances mark a paradigm shift in poultry genomics, positioning the field to close the genotype-molecular-phenotype loop, and equipping the industry with tools to enhance production efficiency, resilience, and animal welfare for long-term sustainability.

## Introduction

Genomic sciences including genomics, transcriptomics, epigenomics, and integrative multi-omics enable the comprehensive study of genetic and regulatory factors underlying complex traits. By interrogating entire sets of biological molecules and regulatory mechanisms, these approaches provide insights into gene function, cellular diversity, and host–microbe interactions that drive phenotypic variation (Karczewski and Snyder, 2018; Dehau et al., 2022). The past decade has witnessed an unprecedented transformation in poultry genomics, supported by rapid advances in sequencing technologies, functional annotation initiatives, and integrative omics approaches. Today, the emphasis has moved beyond reference genome assembly toward translating genomic information into biological context through annotation of regulatory elements, functional gene validation, and fine-scale dissection of genotype–phenotype relationships. The availability of tools such as CRISPR/Cas systems, single-cell transcriptomics, and 3D genome mapping enables researchers to move from statistical associations toward mechanistic insights into complex traits. At the same time, microbiome–host interactions and epigenetic modifications are increasingly recognized as critical layers of regulation shaping poultry health and productivity.

This symposium brings together diverse perspectives on these emerging technologies, highlighting their potential to address pressing industry challenges such as disease resistance, climate resilience, production efficiency, and animal welfare, and to advance precision breeding and sustainable poultry systems. Topics include functional annotation to improve the interpretation of genomic data, CRISPR-enabled discovery of gene function, immune cell census through single-cell technologies, microbiome-driven approaches for health and productivity, and integrative omics to resolve complex traits. Collectively, these presentations provide a forward-looking perspective on how advanced genomics can address critical challenges in poultry production, including disease resistance, environmental sustainability, and animal welfare, while equipping the industry with innovative tools for long-term resilience (Hasin et al., 2017; Gallagher and Chen-Plotkin, 2018; Urgessa and Woldesemayat, 2023).

Unlike previous reviews or research articles, this symposium uniquely integrates perspectives from complementary domains: functional annotation, genome editing, single-cell profiling, microbiome research, and integrative omics, to form a unified framework for precision breeding. The novelty lies in its synthesis of mechanistic tools and validation pipelines that collectively advance poultry genomics toward actionable breeding solutions.

## Functional annotation to support genomic analyses

### Fiona McCarthy, School of Animal & Comparative Biomedical Sciences, University of Arizona

Advances in genome sequencing provide foundational resources for modern genetic analyses in poultry, supporting breeding, gene editing, and the elucidation of genotype-phenotype relationships critical for precision agriculture. Multiple genomes are now available for key avian species, including chicken, duck, goose, and turkey (Dalloul et al., 2010; Hu et al., 2024; Warren et al., 2017). However, the genomic sequence itself is of relatively low informational value, and genome annotation is required to transform sequence into meaningful biological information. Genomic annotation comprises of two complementary approaches: structural and functional annotation.

Structural annotation involves gene identification of functional elements and variation within the genome. While both NCBI and Ensembl annotation workflows identify genes, differences in these workflows will result in overlapping but non-identical gene sets. For example, Ensembl reports 17,007 coding and 13,040 non-coding genes for broiler chicken, while NCBI identifies 18,024 coding and 6,291 non-coding genes. Recently, considerable effort has been focused on experimental annotation to identify regulatory elements in farm animal genomes via the FAANG initiative (Andersson et al., 2015; Clark et al., 2020).

Although associating gene products with functional information was initially done as a distinct and separate process, the development of increasingly accurate sequence-based functional prediction tools means that this process is increasingly included after gene finding. Ensembl provides Gene Ontology (GO) annotation by cross-references and mappings to UniProtKB products which are automatically annotated as part of the Gene Ontology Annotation (GOA) (Huntley et al., 2015) project using InterProScan. The NCBI RefSeq Eukaryote Genome Annotation Pipeline now includes GO term prediction based upon direct application of InterProScan (Goldfarb et al., 2024). However, no equivalent workflow exists to predict function for non-coding gene products.

Standardized gene nomenclature is used to integrate functional annotation. In vertebrates, gene nomenclature includes assigning a gene name based upon known function, a short, unique symbol that represents the full name and identifying any additional gene names used in scientific literature. Standardizing gene nomenclature is the process of ensuring that orthologous genes found in multiple vertebrate species use the same name and symbol to support comparative studies. Gene nomenclature enables unambiguous communication between scientists and supports the integration of data at online databases and tools.

The Chicken Gene Nomenclature Consortium (CGNC) provides standardized gene nomenclature for chicken genes (Burt et al., 2009) that is used by NCBI and Ensembl. Moreover, these genomic resources can propagate gene names and symbols to orthologous genes in other bird species. Our recent work focuses on providing nomenclature for chicken genes not found in humans, or indeed in mammals. The study of these gene sets has not only provided informative gene names but also supported correcting errors in gene annotation and identifying regions of the genome that are misassembled.

The following case studies emphasize approaches for manual annotation of several complex gene sets, emphasizing how applying standardized gene nomenclature supports our understanding of gene function.

• Case Study 1: Standardizing Gene Nomenclature for Poultry MHC Genes.

The major histocompatibility complex (MHC) region in chickens is split into two genetically distinct regions: MHC-B (Kaufman et al., 1999) and MHC-Y (Briles et al., 1993). Both MHC-B and MHC-Y are on chicken microchromosome 16, where they are physically separated by a region of repeats and assort independently. This distinct organization allows for the co-evolution of MHC genes within stable haplotypes and results in strong associations with resistance or susceptibility to various infectious diseases. The MHC-B region contains the classical MHC genes (class I, II, and IV) while the MHC-Y region contains non-classical MHC genes structurally similar to MHC-B genes but differing in their expression levels and functions. As with any MHC region, there is little orthology between species (even if some genes are functional equivalents), and annotation of this region requires manual review. Moreover, this region will vary considerably between lines and individuals, and its contribution to flock health makes it a region of interest for targeted breeding.

**Fig. 1:**
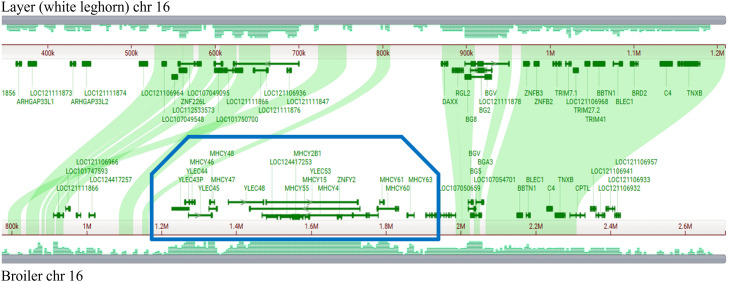
Comparison of broiler and layer MHC regions. Comparison of chromosome 16 from broiler (GCF_016699485.2) and layer (GCF_016700215.2) assemblies indicating the difference in size and missing MHC-Y region in the layer genome. Visualized using the NCBI comparative genome browser (Rangwala et al., 2024), chr 16 is represented by gray horizontal bars, with the layer chr 16 at the top (1.2Mbp) and the broiler chr 16 at the bottom (2.6Mbp). In the middle is a gene view with green shading representing syntenic regions. The boxed region from 1.2 to 1.8Mbp on broiler chr 16 represents the MHC-Y region which is missing from the current layer assembly.

Starting with the broiler MHC-Y genes, we developed a systematic way for naming class I and class II genes, along with other MHC-Y genes commonly found in this region. Genes names include the MHC-Y region designator and can be easily extended as other genes are found in other chicken lines. We extended this system to include the MHC-B region genes which include novel B antigens (from the butryophilin gene family). To test the robustness of the gene naming system, we extended this approach to the layer MHC regions. Close study of the layer MHC identifies that the current assembly (GCF_016700215.2) has a large gap that spans the MHC-Y region (Fig. 1), which is also reflected in the size differences between broiler and layer chromosome 16. Future work will expand this system to the turkey and focus on naming c-type lectins and butryophilins not found in the MHC region.

This case study highlights the need for manual review of complex gene sets and how this process can identify regions of the genome that require improved sequencing and assembly.

• Case Study 2: Expansion of Innate Immune Receptors in Chicken.

Immunoglobulin receptors are a key component of vertebrate immune receptors. Human leukocyte immunoglobulin-like receptors (LILRs) and killer immunoglobulin-like receptors (KIRs) both regulate immune responses (Long et al., 1996; Tedla et al., 2011). Chicken has immunoglobulin receptors which are functionally equivalent to LILRs and have homologous sequences although they do not retain a strict orthologous relationship. Previous studies referred to these genes as “chicken homolog of immunoglobulin-like receptors” or “CHIR”. While gene nomenclature guidelines recommend avoiding species names in gene names, multiple chicken publications used the CHIR designation. To retain this symbol while maintaining best practice we adopted “cluster homolog of immunoglobulin-like receptor” (CHIR) nomenclature, with the CHIRA prefix applied to activating CHIR genes, the CHIRB prefix applied to inhibitory CHIR genes and CHIRAB to designate dual purpose genes (Sparling et al., 2023). A small subset of CHIR genes were insufficiently annotated due to poor sequence or sequence assembly and were provided with the temporary nomenclature “CHIR like” (CHIRL). Future work is ongoing to review additional immune receptors and to expand this nomenclature to other poultry species.

Another type of innate immune receptors are the scavenger receptor (SCAR) genes, so named after initial research identified macrophage receptors involved in clearance of cellular debris and pathogens. In mammals, SCARs are divided into twelve classes of scavenger receptors (designated A-L), based on their protein structure and nucleotide sequence (PrabhuDas et al., 2014). Manual annotation of chicken SCAR genes indicates that the chicken genomes contain many of the SCAR genes identified in humans in addition to gene expansions which account for an additional 22 SCAR genes (Kirkpatrick and McCarthy, 2025). Analysis of selected Sauropsida species indicates that birds and reptiles have more SCAR genes than human, although the number of gene duplication events vary across these species.

This case study demonstrates how systematic review can provide additional functional information in cases where rapid evolution and lineage-specific expansions create functional equivalents.

• Case Study 3: Specialized Repertoire for Environmental Sensing in Poultry.

Vertebrate olfactory receptors (ORs), previously known as odorant receptors, are chemoreceptors expressed in cell membranes which detect small molecules (Niimura, 2009). ORs are a type of G protein-coupled receptors (GPCR) and can be identified by the presence of this domain (Buck and Axel, 1991). However, the OR gene family consists of hundreds of genes, and the gene repertoire varies considerably between species. Since in many cases the OR proteins bind a broad class of molecules or what they bind is unknown, ORs are classified based upon sequence similarity (Olender et al., 2020). The OR gene symbol prefix is “OR” followed by a number designating the subgroup, a letter denoting the subfamily and then a number denoting the specific family member. For example. OR2A1 is olfactory receptor family 2 subfamily A member 1.

We have identified 469 protein coding ORs from chicken (broiler and leghorn), 104 from turkey and 520 from duck (1,093 proteins total). Our initial analysis assigned poultry OR proteins to OR subgroups 2, 4, 5, 6,8 10, 11, 12, 14, 51 and 52. As previously reported, most poultry ORs are assigned to OR14 (Steiger et al., 2009). Our ongoing work will further classify these OR proteins and their corresponding genes, assigning subfamilies and individual members. Since other vertebrate genomes have relatively few OR14 genes, this work will provide systematic nomenclature to unambiguously classify novel poultry ORs. Olfactory receptors and cytochrome P450 genes are used as indicators of adaptive capacity to environmental changes. Future work will focus on reviewing the P450 gene repertoire in the poultry.

• Case Study 4: Novel Genes for Regulating Spermiogenesis in Chickens.

The G2/M-phase Specific E3 ubiquitin protein ligase (G2E3) is a conserved, multifunctional gene that regulates the G2/M transition in the cell cycle through its E3 ligase activity, targeting proteins involved in DNA damage response and mitotic checkpoints for ubiquitin-mediated degradation (Brooks et al., 2007). G2E3 contains both RING and HECT domains, enabling precise substrate selection and degradation. The closely related PHD Finger Protein 7 (PHF7) contains two PHD domains recognizing H3K4me3 histone marks but lacks the HECT domain found in G2E3 (Wang et al., 2017). PHF7 modulates chromatin structure and transcription, playing a key role in spermatogenesis and male fertility. Like other vertebrates, chicken contains a single copy of G2E3 but the PHF7 gene in chicken has 60 gene copies, including 16 genes located on chromosome Z. Other birds show variations in PHF7 gene expansion effect, although this finding may be mitigated by assembly and annotation errors, particularly in the Z chromosome.

The expanded PHF7 gene set was systematically renamed according to their phylogenetic clustering, yielding a standardized and hierarchical nomenclature (e.g., PHF7A1, PHF7A2, PHF7B1). This refined classification provides clarity in a gene family that had previously suffered from annotation ambiguity and highlights the importance of protein domain analysis in gene identification. While phylogenetic tree topology and sequence identity offer valuable insights into evolutionary relationships, domain structure adds a critical layer of functional context. In this case, the HECT domain served as a decisive marker to rule out G2E3 identity, emphasizing the need for a multifaceted approach to gene classification that integrates both evolutionary and biochemical evidence.

## CRISPR-based in vitro approaches for investigating chicken genome function

### Tae Hyun Kim, Department of Animal Science, The Pennsylvania State University

Building upon standardized genomic resources described in the previous section, CRISPR-based functional assays provide the experimental validation needed to confirm gene regulatory mechanisms in poultry. The implementation of genomic technologies in livestock has enabled powerful high-throughput genetic analyses to identify variants related to economically important traits, drastically improving the response to selection. However, translating these statistical associations into mechanistic insights remains a significant challenge. Most (>90 %) of genome-wide significant variants are found in non-coding regions, making the task of linking them to relevant genes or pathways non-trivial (Tak and Farnham, 2015). To address this, the FAANG community has produced comprehensive maps of functional elements and transcripts by generating valuable functional genomics data across different tissues and cell types (Kern et al., 2021; Pan et al., 2023). While these efforts have uncovered tens of thousands of putative regulatory elements in the non-coding regions based on statistical models, the actual biological functions of these elements remain largely unknown and require experimental validation.

**Fig. 2:**
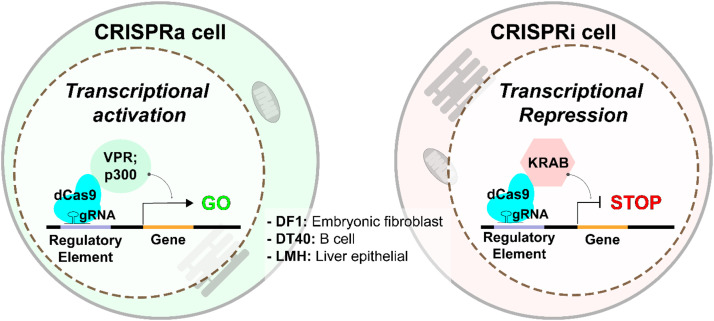
In vitro resources for CRISPR activation (CRISPRa) and CRISPR interference (CRISPRi) in chicken.

The discovery of the Type II Clustered Regularly Interspaced Short Palindromic Repeats (CRISPR)/CRISPR-associated protein 9 (Cas9) system revolutionized biologists’ ability to perform targeted genome editing, making reverse genetics more accessible for functional studies of the genome (Jinek et al., 2012). This technology has since been repurposed for a wide array of new functions, creating a versatile molecular toolbox that extends far beyond simple DNA cleavage. A key innovation was the engineering of a nuclease-deactivated Cas9 (dCas9) that can no longer cut DNA but retains its ability to be guided to a specific genomic address by a guide RNA (gRNA). By fusing different effector domains—proteins with specific functions—to dCas9, the complex is transformed into a programmable, RNA-guided DNA binding platform. This platform can be used to activate (CRISPRa) or repress/interfere (CRISPRi) with gene expression (Fig. 2), or even alter the local chromatin structure to modulate transcription, enabling functional interrogation of any part of the genome in its native context (Dominguez et al., 2016). Further innovations, such as base and prime editing, allow for precise changes to the DNA sequence without inducing double-strand breaks (Anzalone et al., 2019; Komor et al., 2016). Despite the conceptual simplicity and versatility of these tools, their implementation in poultry research has lagged, largely due to challenges associated with the unique embryonic development of avian species and the steep learning curve required to master the technology.

To address these challenges, we developed a streamlined, user-friendly in vitro CRISPR platform tailored for the chicken genome. It is a publicly accessible *in vitro* platform, with accompanying cell lines, plasmids, and protocols available to the research community for broad adoption and collaboration. Our objective was to make these advanced tools accessible, creating a "plug-and-play" system where researchers could manipulate any genomic target with minimal prerequisite steps. We utilized readily available immortalized chicken cell lines representing diverse lineages: DF-1 (embryonic fibroblast), DT40 (B-cell), and LMH (liver epithelial). In each cell line, we engineered stable, constitutive expression of CRISPRa and CRISPRi modalities. For CRISPRa, we have utilized effectors such as the potent VPR transcriptional activator and the p300 histone acetyltransferase to upregulate target gene and locus expression (Hilton et al., 2015; Kiani et al., 2015). For CRISPRi, we fused dCas9 to the KRAB transcriptional repressor to achieve targeted gene silencing (Gilbert et al., 2013). Using a combination of homology-directed repair (HDR) and the piggyBac transposon system, we ensured robust and stable expression of these CRISPR effectors. This design streamlines the workflow; a researcher only needs to design and introduce a gRNA targeting their gene or locus of interest to achieve precise genetic perturbation.

This platform provides a comprehensive toolkit for moving beyond genomic prediction to functional validation, and it is highly effective for dissecting the function of cis-regulatory elements. By targeting gRNAs to promoter regions, we can precisely upregulate or downregulate a target gene, confirming promoter activity and enabling gain- or loss-of-function studies. We demonstrated robust, targeted modulation of multiple genes in DF-1 cells using both CRISPRa and CRISPRi systems (Chapman et al., 2023). The platform is equally powerful for validating distal enhancers. We have shown that by targeting putative enhancers from FAANG data with the CRISPRa system, we can identify their target genes and confirm their regulatory activity, a critical step in understanding the complex landscape of gene regulation (Han et al., 2023). A key application of the platform is its ability to connect statistical signals from genome-wide association study (GWAS) to biological function. As a case study, we used CRISPRa to investigate a non-coding SNP associated with meat flavor (IMP content) located within an intron of the *DUSP8* gene [Kim et al., *accepted*]. Activating this SNP-containing region in DF-1 cells revealed its function as an alternative promoter, driving high expression of a non-canonical, shorter *DUSP8* transcript isoform. Transcriptomic analysis linked this activation to muscle-related pathways, providing a direct molecular mechanism for the GWAS signal. This work demonstrates how a non-coding variant can influence a complex trait by controlling the expression of a specific gene isoform, directly linking a statistical association to a concrete biological function.

The CRISPR-based in vitro platform provides an accessible, robust, and scalable resource for the poultry science community. By lowering the technical barriers to functional genomics, this toolkit can empower researchers to validate regulatory elements, dissect gene function, and translate GWAS findings into actionable biological knowledge. This work bridges a critical gap in poultry science, providing the means to connect genotype to phenotype with unprecedented precision. Future work will focus on expanding this platform to include additional cell lineages and integrating it with high-throughput single-cell based CRISPR screening methods, such as CRISPR droplet sequencing (CROP-seq) or Perturb-seq (Datlinger et al., 2017; Dixit et al., 2016), to dissect complex gene regulatory networks at scale. By making functional genomics more accessible, this platform will help enhance genomic selection, inform precision breeding, and drive new discoveries that contribute to a more sustainable and efficient poultry industry. These resources, including cell lines, plasmids, genomic data, and detailed protocols, are available to the research community to facilitate broad adoption and collaboration.

## A single cell and nuclei review of chicken immune cell types in the thymus

### Wesley C. Warren, Division of Animal Sciences, University of Missouri

While genome editing offers a direct route to functional validation, understanding how genetic variation manifests at the cellular level requires single-cell approaches. The following presentation explores how single-cell and single-nucleus transcriptomics are revealing the complexity of avian immune cell populations, laying the groundwork for dissecting disease resistance and immune resilience. With billions of chickens hatched annually, they remain one of the world’s most essential food-producing animals. Safeguarding the poultry industry against emerging and established viral threats requires a robust genetic strategy designed to minimize, prevent, and respond to rapidly evolving pathogens, particularly fast-spreading viruses. Vaccination is the foremost line of defense against many viral diseases; however, vaccines often lose effectiveness or are less efficacious to begin with. Consequently, there is a crucial need for ongoing genetic refinement of birds that exhibit heightened resilience to viral and bacterial pathogens. Achieving this goal depends on a comprehensive understanding of the genetic and molecular mechanisms underlying disease resilience and resistance. This requires detailed catalogs of immune cell types, their transcriptomic profiles, and their dynamic responses to pathogen challenge. Such insights can strengthen vaccine development, guide selection for polygenic sources of resistance, and identify novel alleles for potential germline edits that improve disease resilience. By building on the success of cell atlas projects in other species, generating high-resolution maps of molecular markers across avian cell types and organ systems will provide an essential resource for linking traits to specific cell populations and for monitoring trait outcomes in future breeding and disease resilience strategies.

**Fig. 3:**
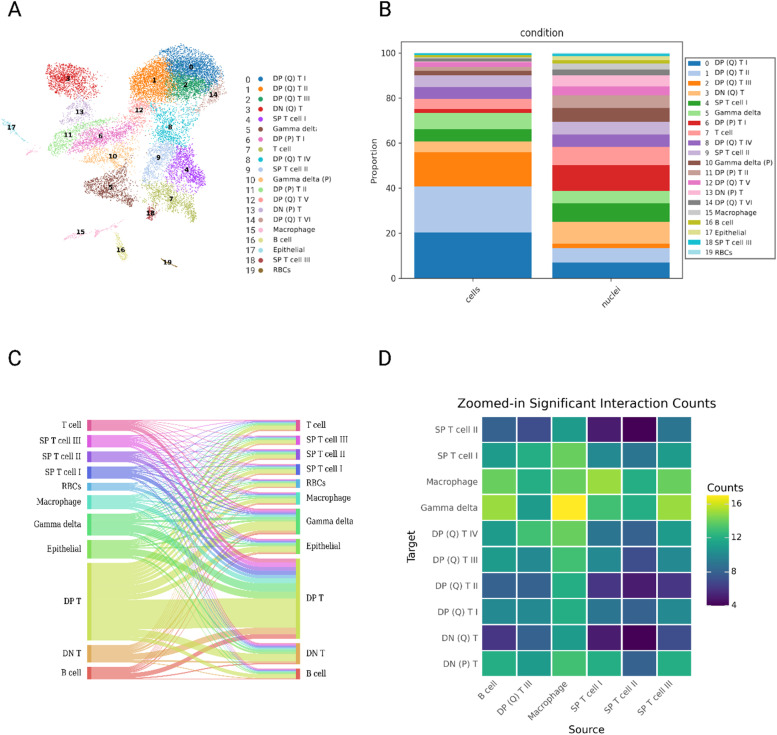
The characterization of cell types in integrated thymus single cell and single nuclei RNAseq data. (A) UMAP visualization of the 20 initial integrated thymus clusters. Of these, all but four clusters were identified as T cells, comprising 94 % of the thymus tissue. (B) a summary revealing differences in cell type proportions across samples, cell types are colored coded and numbered by cell types to match those annotated in panel A. (C) Sankey plot visualizing overall cellular interactions in all cell types present in the thymus dataset. Double positive (DP; CD4^+^, CD8^+^) T cells displayed the highest number of interactions, primarily with other DP T cells. (D) CellPhoneDB heatmap showing an immune cell type focused counts of significant (p value < 0.05) interactions between cell types. Notably, the highest communication was observed between γδ T cells and macrophages (>16 interactions).

Single-cell RNA sequencing (scRNA-seq) has fundamentally transformed our ability to resolve how individual cells, the basic units of life, contribute to phenotype. With the rapid adoption of single-cell technologies, numerous hypotheses have emerged regarding cellular networks that shape pathogen response, protection, and susceptibility in chickens (Shah et al., 2021; Qu et al., 2022; Wu et al., 2023; Warren et al., 2023; Maxwell et al., 2024). The cellular composition of chicken thymus was first described in 1974 (Droege et al., 1974), and since then, identification of major resident cell types, primarily T cells, has relied on low-resolution techniques (Erf et al., 1998; Bucy et al., 1990; Chen et al., 1994). As in all vertebrates, a well-functioning thymus is essential for mounting effective immune responses against infection and disease. Recently, scRNAseq-based characterization of thymic T cell subtypes has changed this perspective, offering new insights into their molecular profiles (von Heyl et al., 2025). In that study CD3^+^ sorted cells, a widely expressed marker for T cells, from the chicken thymus gland at various stages of development revealed the presence of the major T cell types as well as new gene markers that better define the classical γδ and **αβ** lineages. In alignment with these forementioned single cell research initiatives, we have generated molecular profiles of all cell types in the thymus that can connect immune trait selection to cell types that complement existing data sets and extend current efforts for poultry immunogenetics. However, often logistical and biological limitations restrict access to live cell isolates for scRNAseq data generation that immediate tissue cryopreservation can address with subsequent single nuclei RNA sequencing (snRNA-seq) analysis. To evaluate the potential of snRNAseq data interpretation in chicken thymus we integrated scRNAseq and snRNAseq data. A filtered total of 16,301 cells and 893 mean genes detected produced 20 transcriptionally unique clusters of which 94 % were T cells (Fig. 3A). Within sample groupings of cells or nuclei their proportion by cell type was highly similar but also individual biological variability was observed (Fig. 3B). In fact, some T cell populations (not subtype annotated) were significantly less or more prevalent in nuclei or cells by comparison (Fig. 3B). Macrophages were unexpectedly higher in nuclei while epithelial cells were not detected in scRNAseq data. We also note, all nuclei and cells did not colocalize everywhere as observed in other nuclei and cell integration studies (Eraslan et al., 2022; Wu et al., 2019). Our characterization of all cell types in chicken thymus substantially extends this prior thymus cellular knowledge aided by larger numbers of sampled cells, no restrictive cell sorting, inclusion of new T cell markers, and the inclusion of integrated cell and nuclei data sets (von Heyl et al., 2025).

Defining the communicative roles of different T cell subsets in avian immune resilience or resistance to the vast array of pathogens remains challenging, particularly in the absence of baseline communication networks in healthy individuals. To bridge this gap, computational inference of cellular communication has been widely applied to uncover and hypothesize the key modes of cellular crosstalk driving immune function. In humans, such inference approaches have shown strong concordance with complementary data modalities, including intracellular signaling and spatial transcriptomics (Dimitrov et al., 2022; Liu et al., 2022). A central question in avian immunogenetics is therefore to determine which cellular interactions are most critical in shaping immune responses under both healthy and diseased conditions. Many methods now exist to infer how cells are communicating to each other (Armingol et al., 2024). To investigate the intercellular crosstalk and latter downstream signaling pathways in receiver cells we deployed the LIANA+ package on integrated scRNAseq and snRNAseq data (Dimitrov et al., 2024). The inferred analyses of ligand to receptor interactions reveal intricate and previously unappreciated crosstalk between T cells and other cell types (Fig. 3C). A total of 10,525 significant (p value <0.05) ligand to receptor interactions were found that largely comprised DP T populations (Fig. 3C). Frequent communication was observed between γδ T cells and macrophages (>16 interactions: Fig. 3D). Macrophages frequently served as a communication target and source for many cell types. Although more limited in scope, our results reveal striking differences in interactions across cell types, while also highlighting notable commonalities. By inferring cellular crosstalk within the context of a host immune system in healthy birds, this study generates a range of testable hypotheses for how these communication networks may be altered during bacterial or viral infections in chickens.

In conclusion, this avian census of thymic nuclei and cells gene expression profiles of all cell types improves our resolution of T cell subtypes present and their putative crosstalk underlying resilience or resistance to pathogen infection. We identified gene expression signatures and their enrichment in immune-related pathways, providing valuable resources for future research in poultry immunogenetics and disease resistance. While non-immune cell types were scarce in the adaptive thymus, their presence underscores the need for additional data to clarify their often-overlooked roles in communication with immune cells, particularly those involved in antiviral defense, a central threat to poultry health today. Establishing a baseline of cellular communication in the healthy bird thymus is critical for assessing how environmental perturbations shape immune outcomes. Although a systems-level view of avian immune interactions remains an ongoing endeavor, the resources from this study represent an important step toward leveraging natural variation to enhance immune resilience in poultry.

## Microbiome-based strategies for sustainable poultry production

### Guolong Zhang, Department of Animal and Food Sciences, Oklahoma State University

**Fig. 4:**
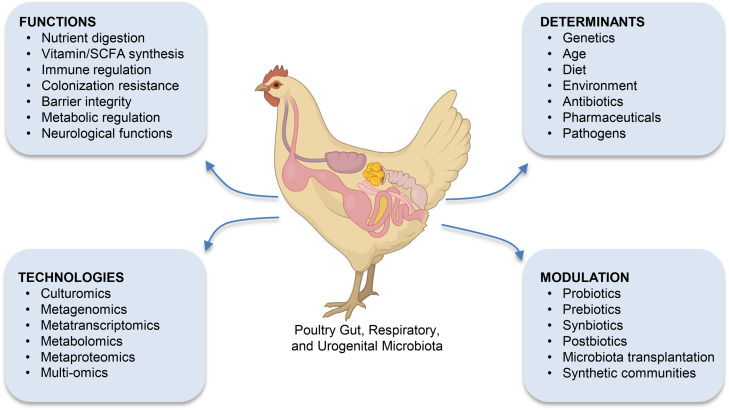
Overview of the major physiological functions, environmental and host-associated determinants, modulation strategies, and analytical technologies related to poultry microbiota.

Insights from immune cell atlases emphasize host-level responses to pathogens, yet poultry health and productivity are also shaped by complex microbial ecosystems. The subsequent section expands the symposium focus from host genetics to host–microbiome interactions, illustrating how microbiome-based strategies complement genomic selection for sustainable poultry production. The microbiotas of the gastrointestinal, respiratory, and urogenital tracts constitute a dynamic ecosystem that profoundly influences poultry health, development, and productivity (Fathima et al., 2022; Rychlik, 2020) (Fig. 4). These microbial communities, consisting of a diverse array of bacteria, archaea, protists, fungi, and viruses, play a central role in host physiology by facilitating digestion, modulating metabolism, shaping immune responses, and supporting tissue development (Hou et al., 2022). In the gastrointestinal tract, microbes assist in the breakdown of complex dietary components, synthesize essential nutrients such as vitamins and short-chain fatty acids (SCFAs), and contribute to energy harvesting. They also promote immune tolerance and protect against pathogens through colonization resistance (Caballero-Flores et al., 2023). Beyond local effects, the microbiome influences neurobiology and behavior via the gut-brain axis, impacting mood, cognition, and stress responses (Mayer et al., 2022). Additionally, microbial metabolism contributes to detoxification and biotransformation of xenobiotics, thereby modulating the host’s response to environmental chemicals and pharmaceuticals. These multifaceted functions underscore the microbiome’s role as a key determinant of animal health and ecological fitness.

Microbiome research has rapidly evolved into a transformative field in sustainable animal agriculture, driven by advances in molecular, cellular, and computational technologies. High-throughput next-generation sequencing enables detailed characterization of microbial communities, while culturomics facilitates the isolation of diverse bacterial species from various mucosal surfaces of chickens (Liu et al., 2023b; Ma et al., 2023). Investigating the roles of individual commensal bacteria in animal health and productivity offers significant potential for identifying promising probiotic candidates for poultry production. Shotgun metagenomics provides comprehensive insights into taxonomic composition and functional potential, and metatranscriptomics reveals actively expressed microbial genes under varying environmental and host conditions (Duan et al., 2024). Complementary platforms such as metabolomics and metaproteomics capture downstream biochemical activities, including metabolites, signaling molecules, and proteins that mediate host–microbe interactions. Integration of these multi-omics approaches offers a systems-level framework for understanding microbial ecosystem dynamics and their impact on host physiology (Zhang et al., 2024). When combined with host transcriptomic, genomic, and epigenomic data, these tools are essential for unraveling the complex interplay between diet, environment, pathogens, and host physiology in poultry.

Host genetics plays a critical role in shaping the composition and function of the gut microbiome, influencing not only which microbial taxa establish but also how they interact with the host (Ryu and Davenport, 2022; Sanna et al., 2022). Different breeds of chickens exhibit distinct gut microbiota profiles (Broadwater et al., 2025; Richards et al., 2019). There is the growing recognition of the microbiome as a heritable trait influencing productivity and health outcomes. GWAS incorporating microbiome data are being explored to improve selection for feed efficiency and disease resilience. For instance, a GWAS study in chickens has identified host genetic variants near genes such as *MTHFD1L* and *LARGE1* that correlate with the abundance of specific cecal bacteria like *Megasphaera* and *Parabacteroides*, which are linked to feed efficiency (Wen et al., 2021). Integrative multi-omics analyses further revealed that host gene expression, particularly in the duodenal mucosa, exerts a stronger regulatory influence on microbial taxa than genomic variants alone, with genes such as *CHST14* interacting with *Lactobacillus salivarius* to modulate fat deposition through bile salt metabolism (Lan et al., 2025). Additionally, studies on microbial inheritance in chickens demonstrated that 94 % of microbial genera were shared across embryos, chicks, and maternal hens, with moderate correlations in microbiota composition between developmental stages, suggesting that host genetic background contributes to the establishment and stability of core microbiota across generations (Ding et al., 2017). These examples highlight that the microbiome is not solely a product of diet and environment but is also shaped by host genetic architecture, opening opportunities for microbiome-informed selective breeding strategies, whereby animals with favorable microbial signatures—such as enhanced digestive efficiency or disease resilience—are selectively propagated.

In poultry, microbial colonization begins immediately post-hatch, with early exposure shaping immune development and resistance to enteric diseases such as necrotic enteritis (Rychlik, 2020). Beneficial taxa, including *Lactobacillus* and SCFA-producing bacteria, dominate the gut and are instrumental in maintaining gastrointestinal integrity and modulating host immunity. Specific microbial profiles have been linked to feed efficiency (Bernard et al., 2024; Liu et al., 2021; Lv et al., 2021; Wen et al., 2021; Zhou et al., 2023), growth performance (Liu et al., 2023a; Xu et al., 2024), and disease resistance (Broadwater et al., 2025) of chickens. For example, *Subdoligranulum*, a bacterial genus closely related to SCFA-producing *Faecalibacerium*, is negatively associated with feed efficiency (Liu et al., 2021) and weight gain of broiler chickens (Liu et al., 2023a). These studies collectively highlight the microbiome’s potential as a target for enhancing animal health production efficiency.

Microbiome manipulation technologies are increasingly being adopted in poultry production to improve health and performance (Naeem and Bourassa, 2025). Probiotics, prebiotics, synbiotics, and microbiota transplantation are among the most widely implemented strategies to enhance gut microbial balance, nutrient absorption, and immune modulation. Probiotics are live microorganisms that confer health benefits when administered in adequate amounts, while prebiotics are selectively fermented substrates that stimulate beneficial microbes. Synbiotics combine both approaches to improve microbial survival and colonization, and microbiota transplantation involves transferring gut microbial communities from healthy donors to restore microbial balance (Rychlik, 2020; Zhao et al., 2025). These interventions not only enhance growth performance and disease resistance but also reduce inflammation and stabilize gut ecosystems under stress. Importantly, they offer alternatives to antibiotics, addressing concerns over antimicrobial resistance while promoting animal welfare and sustainable production.

Recent innovations have further expanded the scope of microbiome-based interventions. Engineered probiotics and designer prebiotics are being developed to selectively stimulate beneficial taxa or metabolic pathways (Chua et al., 2017). Postbiotics—comprising microbial metabolites and structural components—are gaining attention for their stability and reproducibility compared to live microbes (Yue et al., 2025). Synthetic microbial communities, assembled from defined consortia, represent a frontier in microbiome engineering, enabling targeted functions such as pathogen resistance or enhanced nutrient utilization (Jennings and Clavel, 2024). For example, a 10-member bacterial consortium was recently found to significantly enhance growth performance, intestinal epithelial barrier function, microbiota maturation, and disease resistance of chickens (Zhang et al., 2025). These strategies reflect a shift toward precision microbiome management, where functional outcomes can be predicted and tailored to production goals. As the field advances, integration of microbiome science into nutrition, breeding, and management practices is expected to play a pivotal role in shaping resilient and sustainable poultry systems.

While recent advances in microbiome science are promising, several key challenges remain in translating these findings into scalable, practical solutions for poultry production. To be viable in commercial settings, any strategy must be convenient and cost-effective. Moreover, microbiome composition is highly context-dependent—shaped by genetics, diet, environment, and management practices—making reproducibility and standardization difficult. Regulatory frameworks for microbiome-based products are still evolving, and issues of safety, efficacy, and long-term impact require thorough validation. Additionally, the complexity of microbial interactions and host responses calls for robust computational models and longitudinal studies to accurately predict outcomes.

Future research should focus on developing cost-effective microbiome manipulation strategies and predictive microbiome models, identifying microbial biomarkers for health and productivity, and refining microbiome-informed breeding strategies. Advances in synthetic biology and microbial engineering may enable the design of next-generation probiotics with tailored functionalities. Integration of microbiome data into precision farming platforms, supported by real-time monitoring and AI-driven analytics, could revolutionize poultry management. Ultimately, a systems-level understanding of host–microbiome interactions will be essential for harnessing the full potential of microbiome-based strategies in sustainable poultry production.

## Integrative omics in poultry genomics: Unraveling complex traits for enhanced production and health

### Huaijun Zhou, Department of Animal Science, University of California, Davis

The integration of microbial, genomic, and functional data presents new opportunities to understand complex traits in a systems context. The next contribution synthesizes these perspectives by demonstrating how multi-omics frameworks, combining genomics, transcriptomics, epigenomics, and 3D chromatin data, are transforming trait dissection and precision breeding in poultry. Significant progress has been achieved in the genetic improvement of economically important traits such as growth, egg production, and feed efficiency in poultry. However, improving traits with low heritability including disease resistance and meat quality remains a major challenge. Recent studies have suggested that complex traits are influenced by a great number of regulatory variants of small effect (Zhou et al., 2025). Consequently, accurate identification of casual variants is essential to improve genomic selection accuracy. Yet, due to potential linkage disequilibrium among casual variants and nearby genetic markers, it can be difficult to distinguish truly causal alleles from those that are merely associated. Integrating functional information provides a way to prioritize putative casual variants and refine discovery.

The FAANG and GTEx (genotype-tissue expression) efforts on chicken have created powerful resources to support this effort (Pan et al., 2023; Guan et al., 2025; Fang et al., 2025). The chicken regulatory elements atlas aggregated 377 datasets (four histone marks, one CTCF transcription factor, Assay of Transposase Accessible Chromatin sequencing (ATAC-seq), RNA-seq) across 23 tissues in adult stage with annotation of 1.57 millions of regulatory elements including promoters, enhancers and other chromatin states (Pan et al., 2023). It now serves as reference map for functional interpretation of GWAS in poultry. Chicken GTEx further expand these resources by generating millions of regulatory effects on primary expression and post-transcriptional modifications across 28 tissues (Guan et al., 2025). Furthermore, the searchable ChickenGTEx portal (http://chicken.farmgtex.org) catalogues approximately 2.2 million molecular QTL, of which 806,229 are fine-mapped and 1,956 are identified as context-dependent (Hou et al., 2025). In addition, the collection features 257 genome-wide datasets profiling seven epigenetic marks that define 15 chromatin states in 23 tissues. Single-cell transcriptomic data are also available, covering 185,376 expression profiles across 191 cell clusters from nine tissues. Finally, the resource documents 96,386 gene–trait associations linked to 108 traits of economic significance in poultry (Hou et al., 2025).

Efforts to pinpoint causal regulatory variants increasingly rely on a multi-layered strategy that builds from annotation resources toward integrative genomic analysis. Genome-wide association studies in diverse or intercross populations remain the foundation to improve detection of subtle and pleiotropic effects. Linking these variants to downstream genes is facilitated by ChickenGTEx, which provides tissue-matched expression/splicing/alternative QTL (eQTL, sQTL, and aQTL) datasets as well as single- and multi-tissue TWAS across more than 100 traits. Such resources, combined with standard pipelines for colocalization, enhance our ability to identify candidate effector genes and relevant tissues. Beyond simple proximity, advances in 3D genomics including haplotype-resolved Hi-C maps integrated with chromatin and expression profiles will allow assignment of distal enhancers to their target genes and reveal allele-specific regulatory interactions underlying phenotypic differences. Importantly, these integrative findings are beginning to inform genomic prediction models, where incorporating regulatory priors can boost both accuracy and response to selection. Collectively, this progression from annotation to integrative analysis outlines a coherent path toward identifying causal regulatory variants and translating them into biological insights and breeding applications in poultry.

On recent study has integrated multi-breed genome resequencing, GWAS (SNP-, haplotype-, and CCA-based), and transcriptomic profiling across hypothalamus, pituitary, ovary, liver, and abdominal fat in a layer population (Wang et al., 2024) and identified hub candidate genes (HCGs) that regulate several egg-laying phenotypes in chickens. Key HCGs include TFPI2, stimulating GnRH secretion in hypothalamic neurons; CAMK2D, enhancing FSHβ and LHβ secretion in pituitary cells; and OSTN, promoting granulosa cell proliferation and steroid hormone synthesis. The study further employs inter-tissue crosstalk analysis to highlight endocrine factors: APOA4 (a hepatokine) and ANGPTL2 (an adipokine) that communicate with the hypothalamic-pituitary-ovarian axis to elevate egg production. Overall, the findings delineate a coordinated, multi-tissue molecular regulatory network that elucidates the systemic genetic architecture underlying egg-laying performance in poultry (Wang et al., 2024).

Building on the integrative multi-tissue framework that revealed systemic regulatory networks controlling egg-laying performance, recent work has extended these approaches to dissect the temporal and tissue-specific genetic architecture of growth traits in chickens. Zhong et al. (2024) constructs a comprehensive genetic atlas of body weight (BW) by integrating GWAS across 30 developmental stages and five types of molecular QTLs spanning 27 tissues. The analysis demonstrates that chicken growth is a stage-dependent process, with chromosome 1 variants influencing global growth trajectories, chromosome 4 loci regulating growth during mid-development (weeks 8–22), and chromosome 27 variants shaping late growth (weeks 23–72). By integrating Transcriptome-Wide Association Study (TWAS), eQTL/sQTL colocalization, Summary data-based Mendelian Randomization (SMR), and fast eQTL Colocalization (fastENLOC), putative causal variants and candidate genes with stage- and tissue-specific effects identified have provided valuable insights for developmentally targeted genomic selection in poultry breeding (Zhong et al., 2024).

Continuing this progression, Zhu et al. (2025) introduced a 16-generation advanced intercross chicken line, dramatically increasing recombination to fine-map growth traits at near single-gene resolution. Across 75 phenotypes, the study detect 611 QTL for 40 growth traits, including 154 single-gene QTL, then integrate GWAS with chicken FAANG promoter/enhancer annotations (Pan et al., 2023) and GTEx eQTL (28 tissues) (Guan et al., 2025); signals were enriched in strong promoters/enhancers and eQTL, and four frameworks (SMR, fastENLOC, Summary-PrediXcan (SPrediXcan), Summary-MultiXcan (SMultixcan)) prioritized 431 functional genes (Zhu et al., 2025). Putative regulatory variants were identified and functionally validated by dual-luciferase or qPCR assays. For example, a noncoding SNP (rs316348444) boosted ST3GAL4 promoter/enhancer activity in small intestine, and an eviscerated-weight locus harbors an intestinal NCAPG enhancer with allele-specific activity and expression shifts (Zhu et al., 2025). This integrative genomic analysis provides a practical framework for translating GWAS hits into regulatory mechanisms and deployable markers for precision selection in poultry. A complementary application focused on another important trait, abdominal fat in broilers, demonstrates this approach. By integrating 3D genomics, epigenomics, and transcriptomics, Shen et al. (2024) dissected the regulatory architecture of abdominal fat deposition in chickens. Using male chickens divergently selected for high and low abdominal fat, the study constructs a chromatin 3D regulatory network, revealing a noncoding variant (rs734209466) that functions as an allele-specific enhancer through binding of the transcription factor IRF4, it modulates the long-range transcription of IGFBP2 and IGFBP5, influencing preadipocyte differentiation and proliferation (Shen et al., 2024), which provides valuable genetic markers for selective breeding.

In summary, these studies demonstrate that regulatory mechanisms of complex traits are often tissue- and stage-specific. Targeted sampling of the relevant tissue at the appropriate developmental window enhances discovery and colocalization power. Advanced intercross lines, divergent selection populations, and diverse breeds provide complementary resolution and allele diversity, while integration with functional priors (e.g., open chromatin, promoters, enhancers, 3D loops) accelerates the path from association signal to causal variant. Together, these integrative approaches outline a coherent path for transforming genomic discoveries into actionable breeding applications in poultry.

To summarize the symposium’s key themes, the following table provides a concise overview of major technologies, representative tools, and their emerging applications in poultry genomics. Together, these examples highlight how functional annotation, genome editing, single-cell profiling, microbiome research, and integrative omics collectively drive measurable progress toward precision breeding and sustainable poultry production (Table 1).

**Table 1:** Key genomic technologies and its applications in poultry breeding.

Technologies	Example Tools/Resources	Applications in Poultry	Industry Potential
Functional Annotation	FAANG, ChickenGTEx	Identification of regulatory variants	Causal variant discovery
Genome Editing	CRISPRa/i, base editors	Functional validation of GWAS hits	Marker validation and gene function
Single-Cell & Nuclei Profiling	scRNA-seq, snRNA-seq	Immune cell atlas, disease resistance mapping	Vaccine design, resilience breeding
Microbiome Analysis	Shotgun metagenomics, metabolomics	Host–microbe interaction profiling	Feed efficiency, health management
Multi-Omics Integration	RNA-seq, ChIP-seq, ATAC-seq, Hi-C	Prioritize regulatory variants	Genomic prediction

## Conclusions

Collectively, the symposium contributions underscore that poultry genomics is entering a functional era in which annotation, validation, and genomic data integration define the path forward. Genomic sequences are no longer endpoints but foundations for mechanistic discovery, where functional annotation, CRISPR-based interrogation, and single-cell approaches converge to reveal regulatory networks driving key economically import traits. The integration of multi-omics with host–microbe and environmental interactions provide a systems-level view that is essential for improving productivity while safeguarding sustainability. As the field advances, partnerships across academia, industry, and global consortia will be critical to ensure that genomic knowledge translates into actionable breeding strategies and practical applications. The long-term goal is to equip poultry science with a robust toolkit that not only accelerates genetic gain but also strengthens the resilience and sustainability of poultry production worldwide.

Despite rapid progress, several challenges remain in translating genomic discoveries into poultry improvement. Statistical limitations, including reduced power in eQTL mapping and context discordant such as tissue mismatches, can obscure regulatory signals, though resources such as ChickenGTEx and emerging single-cell atlases are beginning to overcome these barriers. Linking distal enhancers to their target genes remains difficult, but integrative 3D chromatin and allele-specific loop maps are showing promise in trait dissection. Continued gaps in functional annotation, particularly under conditions relevant to heat stress, infection, and management environments, underscore the importance of FAANG- and ChickenGTEx-driven community standards to ensure data quality and interoperability. Bridging discovery to breeding decisions also requires careful estimation of effect sizes, pleiotropy, and the integration of regulatory priors into prediction models, a pipeline now being tested in poultry selection contexts.

Looking forward, single-cell and spatial multi-omics, proteome and metabolome QTL mapping, and AI-driven causal inference approaches offer powerful opportunities to enhance the cellular and biochemical underpinnings of complex traits. The field is moving toward an iterative framework using integrative omics to identify candidate variants, validating their function *in vitro* and *in vivo*, and ultimately integrating these signals into genomic selection indices that balance productivity, resilience, and animal welfare for a sustainable poultry future.

Translating these technologies into measurable industry outcomes is now within reach. Integration of regulatory variant mapping with genomic selection can enhance accuracy of prediction. CRISPR-based validation pipelines can help identify causal variants influencing economically important traits such as egg production and growth efficiency. Single-cell immune atlases can aid in the selection of birds with enhanced disease resilience, while microbiome-guided breeding strategies improve feed conversion and welfare indicators. Together, these advances accelerate genetic gain and sustainability.

To sustain progress, the community should prioritize data standardization and interoperability across projects. Harmonizing metadata, adopting shared ontologies, and contributing to global repositories such as FAANG and ChickenGTEx will enhance data reusability. For early-career researchers, engaging in international training networks, open-access annotation efforts, and collaborative benchmarking initiatives provides critical opportunities to build expertise in computational and experimental genomics.

## CRediT authorship contribution statement

**Huaijun Zhou:** Writing – review & editing, Writing – original draft, Conceptualization. **Fiona M. McCarthy:** Writing – review & editing, Writing – original draft. **Tae Hyun Kim:** Writing – review & editing, Writing – original draft. **Wesley Warren:** Writing – review & editing, Writing – original draft. **Guolong Zhang:** Writing – review & editing, Writing – original draft.

## Disclosures

The authors declare that they have no known competing financial interests or personal relationships that could have appeared to influence the work reported in this paper.
